# Initial blood pressure is associated with stroke severity and is predictive of admission cost and one-year outcome in different stroke subtypes: a SRICHS registry study

**DOI:** 10.1186/s12883-016-0546-y

**Published:** 2016-02-29

**Authors:** Chi-Hung Liu, Yi-Chia Wei, Jr-Rung Lin, Chien-Hung Chang, Ting-Yu Chang, Kuo-Lun Huang, Yeu-Jhy Chang, Shan-Jin Ryu, Leng-Chieh Lin, Tsong-Hai Lee, the Stroke Registry in Chang Gung Healthcare System (SRICHS) Investigators

**Affiliations:** Stroke Center and Department of Neurology, Chang Gung Memorial Hospital, Linkou Medical Center and College of Medicine, Chang Gung University, 5 Fu-Hsing St., Kueishan, Taoyuan, 33333 Taiwan; Graduate Institute of Clinical Medical Sciences, Division of Medical Education, College of Medicine, Chang Gung University, Taoyuan, Taiwan; Department of Neurology, Keelung Chang Gung Memorial Hospital, Keelung, Taiwan; Clinical Informatics and Medical Statistics Research Center, Chang Gung University, Taoyuan, Taiwan; Department of Electrical Engineering, College of Engineering, Chang Gung University, Taoyuan, Taiwan; Department of Emergency Medicine, Chiayi Chang Gung Memorial Hospital, Chiayi, Taiwan

**Keywords:** Blood pressure, Stroke, Ischemia, Hemorrhage, Cost, Outcome

## Abstract

**Background:**

To investigate if initial blood pressure (BP) on admission is associated with stroke severity and predictive of admission costs and one-year-outcome in acute ischemic (IS) and hemorrhagic stroke (HS).

**Methods:**

This is a single-center retrospective cohort study. Stroke patients admitted within 3 days after onset between January 1^st^ and December 31^st^ in 2009 were recruited. The initial BP on admission was subdivided into high (systolic BP ≥ 211 mmHg or diastolic BP ≥ 111 mmHg), medium (systolic BP 111–210 mmHg or diastolic BP 71–110 mmHg), and low (systolic BP ≤ 110 mmHg or diastolic BP ≤ 70 mmHg) groups and further subgrouped with 25 mmHg difference in systole and 10 mmHg difference in diastole for the correlation analysis with demographics, admission cost and one-year modified Rankin scale (mRS).

**Results:**

In 1173 IS patients (mean age: 67.8 ± 12.8 years old, 61.4 % male), low diastolic BP group had higher frequency of heart disease (*p* =0.001), dehydration (*p* =0.03) and lower hemoglobin level (*p* <0.001). The extremely high and low systolic BP subgroups had worse National Institutes of Health Stroke Scale (NIHSS) score (*p* =0.03), higher admission cost (*p* <0.001), and worse one-year mRS (*p* =0.03), while extremely high and low diastolic BP subgroups had higher admission cost (*p* <0.01). In 282 HS patients (mean age: 62.4 ± 15.4 years old, 60.6 % male), both low systolic and diastolic BP groups had lower hemoglobin level (systole: *p* =0.05; diastole: *p* <0.001). The extremely high and low BP subgroups had worse NIHSS score (*p* =0.01 and *p* <0.001, respectively), worse one-year mRS (*p* =0.002 and *p* =0.001, respectively), and higher admission cost (diastole: *p* <0.002).

**Conclusions:**

Stroke patients with extremely high and low BP on admission have not only worse stroke severity but also higher admission cost and/or worse one-year outcome. In those patients with low BP, low admission hemoglobin might be a contributing factor.

## Background

Cerebrovascular disease (CVD) is an important global socioeconomic issue due to high disease prevalence, high morbidity, and aging populations worldwide. The direct cost of stroke care is related to the intensity of hospital treatment, in-hospital complication, cardiac disease, stroke severity, and related disabilities, etc. [1, 2]. More and more studies have focused on the cost of stroke care with the purpose to achieve better control of health care expenditure. In Taiwan, over 99 % of the population and nearly all the hospitals are compulsorily enrolled in the National Health Insurance (NHI) system. There is a need to determine the potentially modifiable patient factors that are associated with increased medical costs, which may help to redirect resources to take care of other patients.

Several factors have been proposed to predict the outcome and costs of acute stroke. Blood pressure (BP) is a common outcome predictor in acute ischemic (IS) and hemorrhagic stroke (HS), and elevated initial BP has been reported to be associated with poor short-term and long-term outcomes in acute stroke [3]. Previous studies have found J- or U-shaped correlations between initial BP and outcome in stroke patients [3]. In IS, high BP on admission increases the frequency of early neurological deterioration and predicts unfavorable 90-day outcomes [4]. The cutoff values of high BP in predicting poor outcome or mortality were assumed to be 180–220 mmHg in systolic BP and 120 mmHg in diastolic BP [3, 5]. In HS, high BP during admission increases the risk of hematoma extension and predicts poor clinical outcome and mortality [3, 6]. Previous study noted a threshold of systolic BP over 170–200 mmHg may predict hematoma expansion and neurological deterioration [7]. Low BP during admission has been less extensively studied but is reported to be predictive of poor outcome [5]. Cardiovascular complications could be one factor leading to poor outcome in HS patients with low BP [8–10]. Some stroke-related comorbidities such as physical inactivity, health-care associated infection, and cardiovascular cost may have financial impacts on these patients [11–13]. The other possible reason why extremely low and high BP causes poor outcome needs further investigation, and the correlation between initial BP and admission medical cost has not been addressed. The aim of our study was to assess the correlation of initial BP with admission cost and one-year stroke outcome and study the potentially associated factors in both IS and HS.

## Methods

### Patient enrollment and data collection

Patient data were prospectively recorded and retrospectively analyzed from the Stroke Registry of the Chang Gung Healthcare System (SRICHS). The Human Studies Institutional Review Board in Chang Gung Memorial Hospital at Linkou approved this study. SRICHS is a mature electronic chart-based stroke registry system set up on March, 2007 [14]. The clinical information of patients with the diagnosis of acute IS and HS (ICD9 430–437) was automatically enrolled into this registry and was anonymized and de-identified prior to analysis. Laboratory and imaging reports were auto-downloaded from the hospital information system. Clinical data were collected by the primary care staff and further proofread by the task force to ensure accuracy.

All acute stroke patients registered in the SRICHS between January 1^st^ and December 31^st^ in 2009 were recruited for this study if they were admitted within 3 days after symptom onset in Chang Gung Memorial Hospital, Linkou Medical Center. All patients received brain computed tomographic (CT) scans and/or magnetic resonance imaging (MRI) scans to confirm the clinical diagnosis and were classified into IS and HS groups. Patients were excluded if (1) the time of stroke onset was uncertain; (2) intravascular intervention was carried out during admission; (3) subarachnoid hemorrhages due to aneurysm, arteriovenous malformation, and other vascular anomalies were diagnosed; (4) admission costs were more than $33,333 US dollars (equal to 100,000 New Taiwan dollars; 1 US dollar = 30 New Taiwan dollars). We excluded the patients with exceptionally high costs because most of them were related to social problems (such as prolonged hospital stay due to lack of family support or under lawsuit) and were not associated with the stroke or stroke care treatment per se. The eligible patients were classified into high, medium, and low systolic BP groups (≥ 211, 111–210, ≤ 110 mmHg, respectively) and also diastolic BP groups (≥ 111 mmHg, 71–110 mmHg, ≤ 70 mmHg, respectively) according to the initial BP on admission. Initial BP was defined as systolic BP and diastolic BP on admission which was examined by nurses after bed rest for 5 min. The extremely high BP was defined when systolic BP ≥ 211 mmHg or diastolic BP ≥ 111 mmHg due to increased risk of neurological deterioration, mortality, or disability in these patients [3, 5, 7]. The extremely low BP was defined as systolic BP ≤ 110 mmHg or diastolic BP ≤ 70 mmHg based on the increased risk of cardiovascular complications in such patients [8, 9]. For correlation analysis with admission cost and one-year outcome, BP was further subdivided into systolic BP ≤ 110, 111–135, 136–160, 161–185, 186–210, and ≥ 211 mmHg subgroups and diastolic BP ≤ 70, 71–80, 81–90, 91–100, 101–110, and ≥ 111 mmHg subgroups.

Demographic data (age and gender), heart rate, hemoglobin level, dehydration status, stroke risk factors including hypertension, diabetes mellitus (DM), dyslipidemia, heart disease, cigarette smoking, history of previous stroke, and initial National Institutes of Health Stroke Scale (NIHSS) score on admission were obtained for all recruited patients. Hypertension was defined as previous diagnosis of hypertension or under antihypertensive treatment. DM was defined as fasting blood sugar ≥ 126 mg/dl, previous diagnosis of DM or under hypoglycemic agent and/or insulin treatment. Dyslipidemia was defined as fasting blood cholesterol level ≥ 200 mg/dl, triglyceride level ≥ 150 mg/dl, LDL cholesterol level > 130 mg/dl, HDL cholesterol level < 40 mg/dl, previous diagnosis of dyslipidemia and/or under current lipid-lowering agent usage according to our previous study [15]. Dehydration was defined as blood urea nitrogen to creatinine ratio over 15 (BUN/Cr > 15) and infection was defined as concurrent infections during admission [16]. History of heart disease included atrial fibrillation, coronary artery disease, and/or heart failure. Cigarette smoking was defined as daily smoking for at least 6 months prior to stroke onset.

### Cost and outcome assessment

The direct medical cost of acute stroke care was calculated from the time of admission to that of discharge, including the expense for the personnel, imaging studies, laboratory examinations, medications, and any interventional management in the emergency department, intensive care unit (ICU), and neurology ward with a similar costing method across hospitals in Taiwan. The costs in the rehabilitation department were not included because stroke patients may be assigned different types of post-acute rehabilitation programs in different institutions with a similar length of stay according to the regulations by NHI Administration. The cost data of this study were retrieved by linking the SIRCHS to our hospital-based financial system [16]. However, the medical costs in other institution were unavailable. The long-term outcome of each patient was assessed one year after discharge with modified Rankin scale (mRS) by neurologists or stroke case managers.

### Statistical analysis

Data were analyzed using the Statistical Package for the Social Sciences (SPSS) 17.0 (SPSS, Chicago, IL, USA). Kolmogorov-Smirnov test was initially used to test the normality. When comparing the characteristics of patients with IS and HS, the continuous data were expressed as mean ± standard deviation, and were analyzed using an independent *t* test. Nominal variables were examined by the chi-square test for parametric data. When analyzing the IS and HS patients of different BP subgroups, non-parametric tests were used due to non-Gaussian distribution of these clinical data. The continuous data were expressed as median (quartile 1, quartile 3), and were analyzed using the Kruskal-Wallis test. The Nemenyi-Damico-Wolfe-Dunn test was used for post-hoc comparison. Nominal variables were also examined by the chi-square test. Multivariate linear regression models were further used to analyze the coefficient of factors associated with admission costs. We plotted the correlation of initial BP subgroups with admission cost and one-year mRS score. A *p* value of <0.05 indicated statistical significance.

## Results

### Comparison between IS and HS patients

A total of 1173 IS and 282 HS patients were recruited between January 1^st^ and December 31^st^ in 2009. Compared with HS patients, IS patients had older age of onset (IS vs. HS: 67.8 ± 12.8 vs. 62.4 ± 15.4 years old, *p* <0.001) and higher frequency of DM (30.3 % vs. 21.3 %, *p* <0.001), infection (31.2 % vs. 18.2 %, *p* <0.001), elevated LDL (20.1 % vs. 14.5 %, *p* =0.03) and heart disease (32.7 % vs. 17.4 %, *p* <0.001) (Table 1). Compared with IS patients, HS patients had more initial NIHSS (IS vs. HS: 6.6 ± 7.1 vs. 14.7 ± 13.2, *p* <0.001), initial systolic BP (IS vs. HS: 152.2 ± 23.5 vs. 157.0 ± 30.8 mmHg, *p* =0.01) and diastolic BP (IS vs. HS: 85.5 ± 13.6 vs. 88.2 ± 18.4 mmHg, *p* =0.02), more admission costs (2305.9 ± 3351.3 vs. 3566.5 ± 4380.7 US dollars, *p* <0.001), and worse one-year mRS score (2.2 ± 1.9 vs. 3.4 ± 2.2, *p* <0.001).

**Table 1:** Characteristics of patients with IS and HS

	IS	HS	*p*
	*n* = 1173	*n* = 282	
Demographics			
Age, year	67.8 ± 12.8	62.4 ± 15.4	< 0.001^*^
Gender (F/M)	453/720	111/171	0.82
Stroke severity			
Initial NIHSS	6.6 ± 7.1	14.7 ± 13.2	< 0.001^*^
Stroke outcome			
Admission cost, US dollar	2305.9 ± 3351.3	3566.5 ± 4380.7	< 0.001^*^
Length of stay, day	14.84 ± 15.61	18.61 ± 17.88	0.001^*^
One-year mRS score	2.2 ± 1.9	3.4 ± 2.2	< 0.001^*^
Initial blood pressure			
Systolic BP, mmHg	152.2 ± 23.5	157.0 ± 30.8	0.01^*^
Diastolic BP, mmHg	85.5 ± 13.6	88.2 ± 18.4	0.02^*^
Risk factor of stroke			
Hypertension	737 (62.8 %)	164 (52.8 %)	0.15
Diabetes mellitus	355 (30.3 %)	60 (21.3 %)	< 0.001^*^
Dyslipidemia			
TC ≥ 200 mg/dl	305 (26.0 %)	68 (24.1 %)	0.51
LDL ≥ 130 mg/dl	236 (20.1 %)	41 (14.5 %)	0.03^*^
Cigarette smoking	429 (36.6 %)	88 (31.2 %)	0.09
History of previous stroke	420 (35.8 %)	78 (27.7 %)	0.17
History of heart disease	384 (32.7 %)	49 (17.4 %)	< 0.001^*^
BUN/Cr ≥ 15	421 (35.9 %)	120 (42.6 %)	0.07
Infection	214 (18.2 %)	88 (31.2 %)	< 0.001^*^

Mean ± standard deviation is reported for continuous variables and number (%) for categorical variables. Independent *t* test for continuous variables, and chi-square test for discrete variables. *IS* ischemic stroke, *HS* hemorrhagic stroke, *NIHSS* National Institutes of Health Stroke Scale, *BP* blood pressure, *mRS* modified Rankin scale, *TC* total cholesterol, *LDL* low-density lipoprotein, *BUN/Cr* blood urea nitrogen to creatinine ratio. ^*^ *p* value < 0.05 as statistically significant

### Analyses of IS patients

**Fig. 1:**
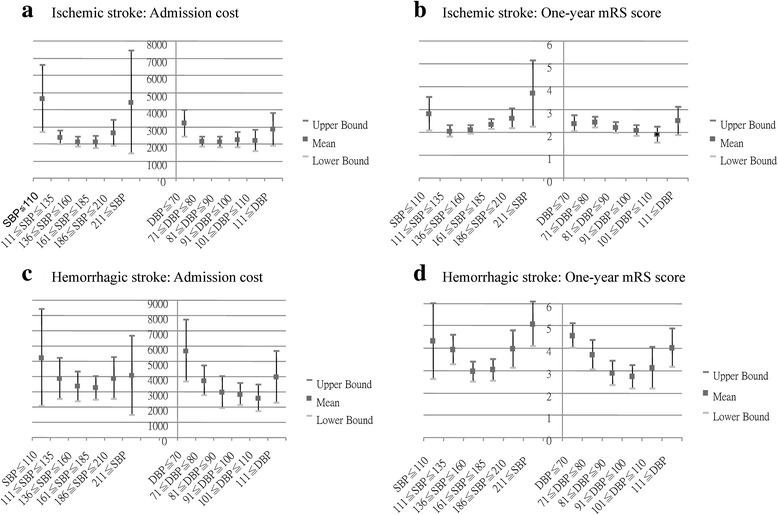
Correlation of admission cost and one-year outcome with initial blood pressure (BP) in patients with ischemic stroke (IS) and hemorrhagic stroke (HS). Correlation of admission cost (**a**) and one-year mRS (**b**) to initial systolic BP (SBP) and diastolic BP (DBP) in IS patients. Admission cost (**c**) and (**d**) one-year mRS score vs. initial SBP and DBP in HS patients

Table 2 shows in the IS patients, high systolic BP group had the highest frequency of hypertension (78.6 %, *p* =0.01). Low systolic BP group had the greatest frequency of infection (*p* =0.03), and low diastolic BP group had the least hemoglobin level (*p* <0.001), the oldest age of onset (*p* <0.001), and the greatest frequency of heart disease (*p* =0.02) among the three BP groups. Compared with the medium BP group, high systolic BP group was male predominant (*p* =0.048) and had worse initial NIHSS score (*p* =0.02). The low systolic BP group had more infection (*p* =0.02), while the high diastolic BP group had younger age of onset (*p* =0.003), and the low diastolic BP group was older (*p* =0.004) and had more heart disease (*p* =0.001) and lower hemoglobin level (*p* <0.001). Dehydration was more common in the low systolic and diastolic BP groups compared with high systolic and diastolic BP groups (*p* =0.04 and 0.03, respectively). When correlated with systolic BP subgroups, admission cost (Fig. 1a, *p* <0.001) and one-year mRS score (Fig. 1b, *p* =0.03) showed a U-shape distribution. Similarly, when correlated with diastolic BP subgroups, admission cost (Fig. 1a, *p* <0.01) also showed a U-shape distribution.

**Table 2:** Analyses of parameters associated with systolic and diastolic BP in patients with acute IS

	Systolic BP (mmHg)				Diastolic BP (mmHg)			
	≤ 110	111 ~ 210	≥ 211	*p*	≤ 70	71 ~ 110	≥ 111	*p*
	*n* = 40	*n* = 1119	*n* = 14		*n* = 154	*n* = 973	*n* = 46	
Age, year	71.5 (56.8,76.5)	69 (59,78)	63.5 (58,77)	0.74	73 (63,80)	69 (58,77)	60.5 (53.3,70.8)	< 0.001^*^
Gender (F/M)	15/25	429/690	9/5	0.14	67/87	368/605	18/28	0.4
Initial NIHSS score	6 (3.3,10)	4 (2,8)	7.5 (3.8,15)	0.03^*^	4 (2,8)	5 (2,8)	5 (2,11)	0.24
Admission cost, USD	1784 (1043.9, 4905.1)	1199.4 (844.5, 1975.7)	2315.0 (1556.7, 5395.3)	< 0.001^*^	1264.5 (893.1, 2556.4)	1201.1 (841.6, 1953.2)	1499.7 (1048.2, 2912.4)	0.01^*^
Length of stay, day	23.5 ± 23.58	14.43 ± 15.06	22.86 ± 21.73	< 0.001	15.53 ± 19.04	14.71 ± 15.1	15.24 ± 13.76	0.820
One-year mRS score	1.5 (1,5.3)	1 (1,4)	5 (1,6)	0.03^*^	1 (1,4)	1 (1,4)	2 (1,4)	0.41
Hypertension, %	40.0	63.4	78.6	0.01^*^	55.2	63.8	67.4	0.1
Diabetes mellitus, %	22.5	30.3	50.0	0.16	24.7	31.0	32.6	0.26
Heart rate, bpm	82 (71.5,89.5)	74 (65,84)	77.5 (73.5, 97.3)	0.002^*^	72 (62,82)	74 (65,84)	85.5 (75.8,98.2)	< 0.001^*^
Previous stroke, %	41.7	37.5	38.5	0.88	35.7	38.0	35.0	0.81
Heart disease, %	37.5	32.7	21.4	0.54	44.8	30.7	34.8	0.02^*^
Hemoglobin, mg/dl	13.9 (11.7, 14.7)	13.8 (12.5, 15)	12.6 (10.6, 14.5)	0.19	13.2 (11.8,14.5)	13.8 (12.6,15.1)	14.1 (13.1,15.8)	< 0.001^*^
BUN/Cr ≥ 15, *n* (%)	18 (51.4)	401 (44.1)	2 (16.7)	0.11	66 (51.6)	343 (43.4)	12 (31.6)	0.07
Infection, *n* (%)	13 (32.5)	197 (17.6)	4 (28.6)	0.03^*^	32 (20.8)	171 (17.6)	11 (23.9)	0.38

Median (Q1, Q3) is reported for continuous variables and number (%) for categorical variables. *BP* blood pressure, *IS* ischemic stroke, *F* female, *M* male, *USD* US dollar, One US dollars = 30 New Taiwan dollars, Previous stroke = history of previous stroke, Heart disease = history of heart disease, *bpm* beats per minute, *NIHSS* National Institutes of Health Stroke Scale, *mRS* modified Rankin scale, *BUN/Cr* blood urea nitrogen to creatinine ratio. ^*^ *p* value < 0.05

The multivariate linear regression (Table 4) revealed that admission cost was associated with initial SBP ≤ 110 mmHg (95 % CI, 55.8 to 2338.6; *p* =0.04), initial NIHSS score (95 % CI, 9.5 to 145.2; *p* <0.001), heart rate (95 % CI, 20.8 to 49.5; *p* <0.001), hemoglobin (95 % CI, −220.3 to −12.4; *p* <0.03), and infections during hospital stay (95 % CI, 967.2 to 2037.3; *p* <0.001) after adjusting for confounding variables including extremely high and low initial systolic BP, extremely low initial diastolic BP, initial NIHSS score, heart rate, hemoglobin, ratio of BUN and creatinine, infection during admission, dyslipidemia with total cholesterol > 200 mg/dl, smoking, and history of previous stroke, heart disease, and hypertension.

### Analyses of HS patients

Table 3 shows that when compared with the medium BP group, high systolic BP, high diastolic BP and low diastolic BP groups had worse initial NIHSS score (*p* =0.01, < 0.001, and < 0.001, respectively). Similar to IS patients, HS patients also had correlation between initial BP and hemoglobin level (systolic BP: *p* =0.05; diastolic BP: *p* < 0.001). When correlated with systolic BP subgroups, a U-shape distribution was seen in one-year mRS score (Fig. 1d, *p* =0.002) but not in admission cost (Fig. 1c, *p* =0.29). There was also a U-shape distribution of diastolic BP subgroups with admission cost (Fig. 1c, *p* =0.002) and one-year mRS score (Fig. 1d, *p* <0.001). The multivariate linear regression (Table 4) revealed that admission cost was associated with initial NIHSS score (95 % CI, 0.02 to 78.50; *p* =0.05), and infection during hospital stay (95 % CI, 892.84 to 2947.16; *p* <0.001) after adjusting for confounding factors including extremely low initial diastolic BP, initial NIHSS score, heart rate, hemoglobin, infection during admission and history of hypertension.

**Table 3:** Analyses of parameters associated with systolic and diastolic BP in patients with acute HS

	Systolic BP (mmHg)				Diastolic BP (mmHg)			
	≤ 110	111 ~ 210	≥ 211	*p*	≤ 70	71 ~ 110	≥ 111	*p*
	*n* = 12	*n* = 254	*n* = 16		*n* = 47	*n* = 208	*n* = 27	
Age, year	64.5 (51.5,70.8)	63 (52,75)	54.5 (48.3,64.5)	0.19	74 (58,80)	62 (52,73)	52 (45,62)	< 0.001^*^
Gender (F/M)	5/7	101/153	5/11	0.79	24/23	80/128	7/20	0.09
Initial NIHSS score	19 (8,40)	9.5 (4,19)	24 (14,31)	0.01^*^	16 (8,40)	9 (3.3,16)	16 (10.3,30.8)	< 0.001^*^
Admission cost, USD	2755.6 (2169, 7995.5)	1845.9 (1110.8, 3986.0)	1768.6 (1137.1, 6211.4)	0.29	2629.1 (1596.6, 6628.8)	1668.6 (983, 3649)	1981.2 (1488.4, 4241.6)	0.002^*^
Length of stay, day	13.92 ± 9.55	18.98 ± 17.97	16.19 ± 21.28	0.543	19.68 ± 16.66	18.17 ± 18.23	20.07 ± 17.69	0.790
One-year mRS score	6 (3,6)	3 (1,5)	6 (5,6)	0.002^*^	5 (4,6)	3 (1,5)	5 (2,6)	< 0.001^*^
Hypertension, %	41.7	60.6	31.3	0.03^*^	48.9	61.1	51.9	0.25
Diabetes mellitus, %	33.3	20.5	25.0	0.53	34.0	18.8	18.5	0.54
Heart rate, bpm	83 (77.5,92)	76 (66,86)	88.5 (77.3, 96.3)	0.01^*^	77 (65.5,87.5)	76 (67,87)	83 (73,93.5)	0.23
Previous stroke, %	30.0	32.9	37.5	0.94	41.0	31.3	31.8	0.5
Heart disease, %	25.0	16.9	18.8	0.76	21.3	17.3	11.1	0.54
Hemoglobin, mg/dl	11.8 (10.7,13.6)	13.9 (12,15)	14.0 (12.6,15.6)	0.05^*^	12.7 (11.0,14.0)	`13.9 (12.2,15)	15.2 (13.4,16.2)	< 0.001^*^
BUN/Cr ≥ 15, *n* (%)	6 (54.6)	111 (51.9)	3 (25.0)	0.19	25 (58.1)	85 (49.7)	10 (43.5)	0.47
Infection, *n* (%)	5 (41.7)	77 (30.3)	6 (37.5)	0.62	19 (40.4)	61 (29.3)	8 (29.6)	0.33

Median (Q1, Q3) is reported for continuous variables and number (%) for categorical variables. *BP* blood pressure, *HS* hemorrhagic stroke, *F* female, *M* male, *USD* US dollar, One US dollars = 30 New Taiwan dollars, Previous stroke = history of previous stroke, Heart disease = history of heart disease, *bpm* beats per minute, *NIHSS* National Institutes of Health Stroke Scale, *mRS* modified Rankin scale, *BUN/Cr* blood urea nitrogen to creatinine ratio. ^*^ *p* value < 0.05

**Table 4:** Multivariate linear regression analyses of factors associated with admission costs in stroke patients

	IS				HS			
Admission cost, USD	Factors	Linear regression coefficient	95 % CI	*P* value	Factors	Linear regression coefficient	95 % CI	*P* value
	Initial NIHSS score	112.4	79.5 to 145.2	<0.001	Initial NIHSS score	39.26	0.02 to 78.50	0.051
	Heart rate, bpm	35.1	20.8 to 49.5	<0.001	Infection	1920.00	892.84 to 2947.16	<0.001
	Hemoglobin, mg/dl	−116.4	−220.3 to −12.4	0.028				
	Infection	1502.3	967.2 to 2037.3	<0.001				
	Low SBP (≤110 mmHg)	1197.2	55.8 to 2338.6	0.040				
	High SBP (≥ 211 mmHg)	1763.8	−8.4 to 2526.0	0.051				

*IS* ischemic stroke, *HS* hemorrhagic stroke, *USD* US dollar, *bpm* beats per minute, *NIHSS* National Institutes of Health Stroke Scale, *SBP* systolic blood pressure

## Discussion

Different to previous studies, our results disclosed that in both IS and HS, patients with extremely high and low diastolic BP had greater admission cost. Moreover, we found that low hemoglobin level, presence of infection, severe stroke, and increased heart rate could be the main contributors to the greater admission cost in stroke patients with extreme BP. The modification of these factors may help to reduce the medical expense in the future.

### The extremely low BP and admission costs

Similar to previous reports [4, 17, 18], our results also provided evidence that extremely high and low systolic BP were associated with poor one-year outcome in acute IS and HS patients, demonstrating a U-shape relationship between initial BP and outcome [3, 19]. Furthermore, we noted both our IS and HS patients with low initial diastolic BP had lower hemoglobin level and greater admission cost than patients without these conditions. Hemoglobin level was inversely associated with admission costs but not one-year mRS after adjusting for confounding variables in IS patients. In contrast, our HS patients with low initial diastolic BP had lower hemoglobin and more admission cost, but the correlation between hemoglobin and admission cost was insignificant after adjusting for confounding factors. The plausible explanation is that diastolic BP could be influenced by blood volume [20], and anemia may cause insufficient cerebral blood flow resulting in reduced oxygen-carrying capacity [21]. The low hemoglobin level was previously reported to lead to high cardiovascular and non-cardiovascular mortality in patients with old age, acute coronary syndrome, heart failure, or life-threatening illness [22–25], and could cause prolonged ICU stay and mechanical ventilator use in IS patients [23]. All these conditions may lead to more acute care costs.

The low BP during admission could be secondary to heart failure, coronary heart disease, dehydration, or sepsis [5, 26]. Heart diseases including atrial fibrillation, ischemic heart disease, and congestive heart failure may result in reduced cardiac output, and increased length of hospitalization and inpatient cost in patients with transient ischemic attack or who are dependent [27, 28]. It is possible that patients with incident heart disease are frail and prone to have worsening heart failure during admission [29, 30]. Dehydration may cause low BP, high admission infection rate, and poor clinical outcome [16]. Concurrent infection and sepsis is also known to prolong hospital stay and increase medical cost especially in insured patients, such as our patients under a universal health insurance system [31, 32]. Our IS patients with low diastolic BP had more heart disease and dehydration, and those with low systolic BP had more infection (Table 2). After adjusting for the confounding factors, our study provided evidence that infection could be a major contributor to increased admission costs in patients during the acute treatment of stroke (Table 4).

### The extremely high BP to admission costs

Early intensive BP control in HS was reported to improve outcomes in INTERACT 2 and ATACH-II trials [33, 34]. Our observational study was consistent with the results demonstrating a linear association between physical dysfunction and baseline systolic BP in INTERACT 2 trial [35]. The elevated BP during acute stroke could be related to previous history of hypertension, increased intracranial pressure, stress of hospitalization, or autonomic system activation [26, 36, 37]. The Cushing reflex (raised BP, reduced heart rate, and irregular breathing) which is a CNS homeostatic response to increased intracranial pressure [38] could occur in intracerebral hemorrhage or large ischemic stroke. Both our IS and HS patients with extremely high BP had increased HR instead of bradycardia. It is possible that acute stroke may induce sympathetic activation or development of Cushing reflex which contributes to extremely high BP in the beginning [38]. It is known that high admission blood sugar is associated with poor outcome [39], and our study showed the DM was more common in IS patients with high BP (*p* =0.05) than low BP.

### Initial stroke severity and admission costs

Our results suggested that initial stroke severity is the important contributor to admission cost in IS and HS patients with initial extreme BP (Table 4). Besides, our IS patients with extreme systolic BP and HS patients with extreme diastolic BP had more admission cost and worse one-year outcome especially when associated with worse initial NIHSS score. In previous studies, it has been shown that HS patients with worse initial stroke severity may have longer hospital stay and more post-stroke infection [13, 40]. It is also reported that high baseline NIHSS score is likely to be associated with large infarct volume in patients with anterior circulation stroke and can predict progression of neurological deficit and poor 3-month clinical outcome in patients with posterior circulation stroke [41, 42].

### Limitations

First, our study only included the direct medical costs of acute care but did not collect the costs up to 12 months to correlate with the long-term outcome. Second, since this is a single center study, the generalizability may be limited. However, Chang et al. reported that similar to previous results, the initial stroke severity may predict costs of acute care in Taiwanese IS patients [2]. Since Taiwan has one-payer insurance system that covers more than 99 % of the population, the results of our study could be well generalized in Taiwan. However, the generalizability beyond Taiwan may need further validation because of the diversity of medical insurance system among different countries. Third, we used the initial BP but not the 24-h ambulatory BP as the predictor. The initial BP may be influenced by many factors and is sometimes unreliable. Fourth, we did not consider imaging findings such as infarction/hematoma location and volume, as infarction/hematoma expansion with brain stem destruction may result in low initial BP and is associated with high admission cost and poor stroke outcome. Fifth, we did not make a distinction between infection on admission and in-hospital infection. Finally, the small sample size of our patients with extreme BP may also have limited power to show the significance. Nevertheless, our study has provided evidence that future studies should be undertaken to examine whether the correction of these factors may help reduce the medical expense related to acute hospital care and improve the long-term outcome.

## Conclusions

A U-shape relationship can be seen between initial systolic/diastolic BP and admission cost as well as one-year mRS score in both IS and HS patients. Infection is associated with high admission costs in both IS and HS patients. Severe stroke, increased heart rate, and low hemoglobin and systolic BP on admission significantly increased total admission costs and patients with these conditions should be carefully monitored to avoid complications.
